# The *ham-5*, *rcm-1* and *rco-1* genes regulate hyphal fusion in *Neurospora crassa*

**DOI:** 10.1099/mic.0.040147-0

**Published:** 2010-09

**Authors:** Mash'el Salman Aldabbous, M. Gabriela Roca, Angela Stout, I-Ching Huang, Nick D. Read, Stephen J. Free

**Affiliations:** 1 Department of Biological Sciences, Cooke Hall Room 109, University at Buffalo, Buffalo, NY 14260, USA; 2 Fungal Cell Biology Group, Institute of Cell Biology, Rutherford Building, University of Edinburgh, Edinburgh EH9 3JH, UK

## Abstract

Mutants of *Neurospora crassa* unable to participate in vegetative hyphal fusion (anastomosis) were isolated and characterized. From this analysis, three genes, *rcm-1*, *rco-1* and *ham-5*, were identified and shown to be required for hyphal fusion. The *rcm-1* and *rco-1* genes are homologues of the *Saccharomyces cerevisiae SSN6* and *TUP1* genes, which encode a dimeric transcription factor in yeast. We demonstrate that in *N. crassa* the *rcm-1* and *rco-1* genes are required for hyphal fusion and normal hyphal morphology, and influence both asexual and sexual development. The *ham-5* gene encodes a 1686 amino acid protein with two putative WD40 domains, which might participate in protein–protein interactions. *ham-5* deletion mutants had a reduced rate of hyphal extension and altered hyphal morphology, and were unable to produce the conidial anastomosis tubes that are required for hyphal fusion during colony initiation.

## INTRODUCTION

Cell fusion events play very important roles during the life cycles of *Neurospora crassa* and other filamentous fungi. In the vegetative phase of *N. crassa*, hyphal fusion occurs during colony initiation by means of specialized hyphae called conidial anastomosis tubes (CATs) derived from conidia or germ tubes. These hyphae grow chemotropically towards other CATs and fuse with each other to establish a network of conidial germlings (Roca *et al.*, 2005a, b; Read *et al.*, 2009, 2010). Later on during colony development, hyphal fusion becomes localized in a subperipheral region behind the growing front of the spreading colony. This involves specialized secondary hyphae, called fusion hyphae, that are produced as branches from the vegetative hyphae and grow in a directional manner towards each other, or towards other vegetative hyphae, and fuse to generate an interconnected hyphal network in the mature colony (Fleissner *et al.*, 2008; Hickey *et al.*, 2002; Read *et al.*, 2009, 2010). Hyphal fusion allows the interconnected hyphae of the colony to operate as a coordinated individual and to regulate its overall homeostasis by the interchange of nutrients, water, signalling molecules and cellular organelles (Fricker *et al.*, 2007; Read *et al.*, 2009, 2010).

Cell fusion events are also important during the sexual part of the life cycle of *N. crassa* (Read *et al.*, 2010). In this fungus, which is heterothallic, mating occurs between opposite mating types and commonly involves the fusion of a specialized hypha called a trichogyne (formed from the female protoperithecium) and a male cell that is commonly a conidium (Bistis, 1981; Kim & Borkovich, 2004; Read *et al.*, 2010). After migrating down the trichogyne into the protoperithecium, the male nuclei pair up with the female nuclei to form a dikaryon in the ascogenous hyphae. The binucleate dikaryotic cells of the ascogenous hyphae are maintained by conjugate nuclear divisions accompanied by a specialized form of cell fusion involving the fusion of crozier cells with subapical cells of the ascogenous hyphae. The two nuclei of the dikaryotic cell subsequently fuse to generate a diploid cell that immediately undergoes meiosis to generate the haploid ascospores (sexual spores) within the ascus (Raju, 1980; Read *et al.*, 2010).

Although cell fusion is critically important in the life cycles of filamentous fungi, little is known about the mechanism(s) involved in achieving cell fusion and how cell fusion events are regulated. One important approach has been to identify mutants that are defective in hyphal fusion and to characterize the affected genes in such mutants. In this study, we isolated mutants of *N. crassa* compromised in hyphal fusion and identified three genes (*rco-1*, *rcm-1* and *ham-5*) that are required for this process. RCO-1 and RCM-1 are homologues of the *Saccharomyces cerevisiae* TUP1 and SSN6 proteins, respectively (Kim & Lee, 2005; Yamashiro *et al.*, 1996). In yeast, the SSN6/TUP1 dimeric transcription factor functions to regulate yeast cell growth and development (Keleher *et al.*, 1992; Redd *et al.*, 1997). The formation of CATs, CAT fusion, and hyphal fusion in the mature colony are shown here to be dependent upon the *rco-1* and *rcm-1* genes in *N. crassa*. The third hyphal fusion mutant we identified is affected in the *ham-5* gene. The gene encodes a 1686 amino acid protein that is a close homologue of the *Podospora anserina* *IDC1* gene, which has been shown to be required for NADPH-oxidase-dependent movement of a MAP kinase into the nucleus (Jamet-Vierny *et al.*, 2007). Close homologues of the *IDC1* and *ham-5* genes are found in the genomes of other sequenced filamentous fungi. The HAM-5 protein has two putative WD40 domains, suggesting that it functions as part of a multimeric protein complex involved in regulating hyphal fusion.

## METHODS

### Strains and culture conditions.

*N. crassa* strains *arg-12* a (FGSC 1527), *inl* a (FGSC 1438), *pyr-3*; *ff* (PB-S8) (FGSC 3086), deletion mutants for *ham-5* (FGSC 15045 and 15046), deletion mutants for *rco-1* (FGSC 11371 and 11372), and the repeat-induced point mutation (RIP)-generated *rco-1* mutant (FGSC 9511) were obtained from the Fungal Genetics Stock Center (Kansas City, MO, USA). The strains used in classical mapping experiments, *arg-5 aro-3* A (FGSC 7194), *arg-5 thr-2* A (FGSC 7192), *pyr-4 arg-12* (FGSC 2998) and *cys-3 arg-5* (FGSC 4149) were also obtained from the Fungal Genetics Stock Center. Strains were cultured on Vogel's sucrose medium (hereafter referred to as Vogel's medium) with appropriate supplements (Davis & DeSerres, 1970).

### UV mutagenesis.

The MSA-128 mutant was isolated in a screening experiment designed to generate mutants affected in vegetative hyphal fusion. Mutagenesis experiments were carried out as previously described (Bowman *et al.*, 2006), using conidia from the *arg-12* mutant strain. Mutagenized conidia were plated onto sorbose agar medium supplemented with arginine and individual isolates were picked into test tubes containing Vogel's medium supplemented with arginine. The individual isolates were then tested for their ability to participate in hyphal fusion using the heterokaryon formation assay.

### Heterokaryon formation assay.

For the isolation of hyphal fusion mutants, a heterokaryon-based screening assay was used. Conidia from the individual isolates obtained in the mutagenesis experiment were used to inoculate a test tube containing a slant of non-supplemented growth medium that had previously been inoculated with conidia from an inositol auxotrophic mutant. Isolates from the mutagenesis that were able to form a heterokaryon with the inositol auxotroph, as demonstrated by healthy vigorous growth within 24 h, were discarded. Isolates that did not form a heterokaryon with the inositol auxotroph until 72 h post-inoculation were considered putative fusion mutants and were subjected to further analysis.

In addition to obtaining mutants from mutagenized conidial isolates with the heterokaryon-formation-based screening procedure, we also identified fusion mutants that had been previously placed in the Fungal Genetics Stock Center as protoperithecia-defective mutants. These mutants were examined using the hyphal fusion assays described below. Isolates that were unable to participate in cell fusion as defined by these assays were considered as being fusion-defective.

### Microscopy-based hyphal fusion assays.

Two additional microscopy-based assays were used to assess whether a mutant isolate was defective in the process of hyphal fusion. One of these assays involved identifying by differential interference contrast (DIC) microscopy the flow of cytoplasmic organelles through vegetative hyphal fusions in the mature colony. A cellophane disc was placed over solid Vogel's medium (with appropriate supplementary nutrients) in a Petri dish, and conidia were used to inoculate the middle of the disc. After allowing the conidia to germinate and grow overnight, a second cellophane disc was placed on top of the first one to create a ‘sandwich’ in which the growing colony was trapped between the two cellophane layers. The colony was allowed to continue growing for another hour or more and then a 2 cm square containing the growing edge of the colony sandwiched between the cellophane layers was cut out with a sterile razor blade. The 2 cm square cellophane–colony edge–cellophane sandwich was placed on 50 μl liquid Vogel's medium in a 50 mm Fluorodish (World Precision Instruments) and examined with an inverted DIC microscope. Video recordings of the flow of cytoplasmic organelles through connected fusion hyphae were made and used as a record of the analysis. It was important to position the second cellophane layer at a 9 ° angle to the first in order to minimize detrimental optical effects on the DIC image.

The second microscopy-based hyphal fusion assay involved the identification of CATs during colony initiation according to diagnostic features previously described (Roca *et al.*, 2005a, b; Read *et al.*, 2009, 2010). Briefly, conidia from 4–5-day-old cultures were harvested in growth medium and diluted to a titre of 10^6^ conidia ml^−1^. A 200 μl sample of the conidia was then placed in a well of an eight-well slide chamber (Nalge Nunc International), and the chamber was incubated at 34 °C for 4 h to allow the conidia to germinate and form CATs. The chamber was carefully removed from the incubator and examined with an inverted DIC microscope. CATs were distinguished from germ tubes by being thin and short hyphae that homed towards other CATs and fused to form an interconnected network of germinating conidia. Low-temperature scanning electron microscopy was carried out as previously described (Roca *et al.*, 2005a).

### PCR amplification and identification of mutated genes.

To identify mutations within the genomic DNA of the mutant isolates, the location of each of the mutations was determined by classical genetic analysis and mapping experiments (Davis & DeSerres, 1970). We then examined the annotated genes in the identified genomic DNA regions and prepared lists of candidate hyphal fusion genes. We designed PCR primers to amplify a series of 1200–1400 bp regions of genomic DNA covering these candidate genes and their flanking sequences. The genomic DNA sequence of *N. crassa* published at the Broad Institute was used to identify candidate genes and design these primers (http://www.genome.wi.mit.edu; Dunlap *et al.*, 2007). Primers were obtained from IDT (Coralville, IA, USA). PCR products were amplified, subjected to agarose gel electrophoresis, and cut from the agarose gels. The amplified DNA was purified and sequenced from both ends using the amplification primers (Retrogen). Sequences from the amplified genomic DNAs were then compared with the sequence from the published *N. crassa* genome to identify mutations.

### Assessing gross morphology and development.

To assess the gross colony morphology of each strain, small inocula of each of the mutants and the wild-type were made in the centre of Petri dishes containing Vogel's agar medium. All strains were allowed to grow at room temperature for 72 h and images of the plates were then captured using a SnapScan 1236 digital scanner (Agfa).

To analyse hyphal morphology, all strains were grown at room temperature. The growing edge of the colony was photographed using a Canon Power Shot A620 digital camera with an adaptor for microscope use from Zarf Enterprises. Mycelial extension rates were measured on Petri dishes with solid medium at 25 °C. These extension-rate experiments were carried out in triplicate and the mean extension rates were calculated.

### Protein extraction and Western blot analysis to assess the phosphorylation of the MAK-1 and MAK-2 activation sites.

Conidia from 5-day-old cultures were harvested into sterile distilled water and 10^7^ conidia were used to inoculate cellophane disks that were placed on top of 30 ml of Vogel's agar medium in a Petri dish. The Petri dishes were incubated at 25 °C, and fungal mycelium was harvested 18–36 h post-inoculation. The harvested mycelium was ground into a fine powder with a mortar and pestle in liquid nitrogen, and extraction buffer (50 mM HEPES pH 7.5, 2 mM EGTA, 2 mM EDTA, 1 % Triton X-100, 10 % glycerol, 100 mM NaCl, 1 mM phenylmethylsulfonyl fluoride) and phosphatase inhibitors (1 mM sodium orthovanadate and 1 mM sodium fluoride) were added to the frozen ground fungal mycelium. The frozen mixture was allowed to thaw on ice, subjected to centrifugation (4000 ***g*** for 30 min), and the supernatants were transferred to clean tubes. The concentrations of protein in the extracts were determined using a Bio-Rad protein assay kit.

To carry out the immunoblot analysis, 60 μg total protein (per lane) was separated on an SDS-10 % polyacrylamide gel by electrophoresis. Protein was transferred onto nitrocellulose membranes by wet electroblotting (Bio-Rad) and visualized with Ponceau S red dye (Sigma) to verify that the protein was transferred to the nitrocellulose membrane and that the lanes contained equal amounts of protein. Membranes were rinsed several times with double-distilled water and then blocked for 4 h by shaking in Tris-buffered saline (50 mM Tris pH 7.5, 150 mM NaCl), 0.1 % Tween 20 and 5 % nonfat dried milk (Nestlé Carnation) at room temperature. To detect and distinguish the phosphorylated forms of the two *N. crassa* MAP kinases MAK-1 (51 580 Da) and MAK-2 (41 080 Da), membranes were incubated with anti-phospho p44/42 MAP kinase (1 : 1000 dilution) (PhosphoPlus antibody kit; Cell Signaling Technology) in Tris-buffered saline (50 mM Tris pH 7.5, 150 mM NaCl), 0.1 % Tween 20 and 1 % nonfat dried milk with shaking overnight at 4 °C. The primary antibody (PhosphoPlus antibody kit) was raised against the amino acid sequence HDHTGFLTEYVATRWYRA, which is the sequence at the activation site of the human ERK1 protein. MAK-1 and MAK-2 are both recognized by this antibody (Pandey *et al.*, 2004; Maerz *et al.*, 2008). Both of these proteins have the amino acid sequence TEYVATRWYRA as the carboxyl half of their activation sites (the underlined threonine and tyrosine residues are phosphorylated). After labelling with primary antibody, the blots were washed three times for 5 min each in Tris-buffered saline and 0.1 % Tween 20. Membranes were then incubated with horseradish-peroxidase-conjugated secondary antibody (1 : 2000 dilution) (PhosphoPlus antibody kit) for 1 h at room temperature. Positive and negative controls (phosphorylated and non-phosphorylated p42 MAP kinase (Erk2) were provided by Cell Signaling Technology. The antibody complex was visualized by using an enhanced chemiluminescence PhosphoPlus antibody kit (Cell Signaling Technology) and an ECL kit (Amersham) according to the manufacturers' instructions.

## RESULTS

### Isolation of MSA-128, a hyphal-fusion-defective mutant

When two populations of auxotrophic conidia are placed on a medium without supplementation, they rapidly participate in hyphal fusion and form a prototrophic heterokaryon, which can be identified within 24 h post-inoculation. Pilot experiments with conidia from *so*/*ham-1* and *ham-2* mutants (Wilson & Dempsey, 1999; Fleissner *et al.*, 2005; Xiang *et al.*, 2002), two previously identified fusion defective mutants, showed that *so/ham-1* and *ham-2* conidia required about 72 h before a heterokaryon was formed. We used the inability to rapidly form heterokaryons between two compatible auxotrophic mutants as the basis of a screening procedure to isolate hyphal fusion mutants. Conidia from an *arg-12* auxotroph were mutagenized by exposure to UV light, and 1000 isolates from the mutagenesis were individually picked into test tubes containing a slant of growth medium supplemented with arginine. Conidia from these isolates were then used to inoculate a second growth tube containing unsupplemented medium that had been inoculated with conidia from an inositol-requiring isolate. In almost all cases, the germinating conidia from the two isolates underwent fusion to generate a healthy heterokaryon. Growth of these heterokaryons was readily evident 24 h post-inoculation. We identified 32 mutant isolates that did not show significant growth until 72 h post-inoculation, and designated these mutants as putative fusion-defective mutants. MSA-128 was one of these putative fusion-defective mutants.

**Fig. 1.**
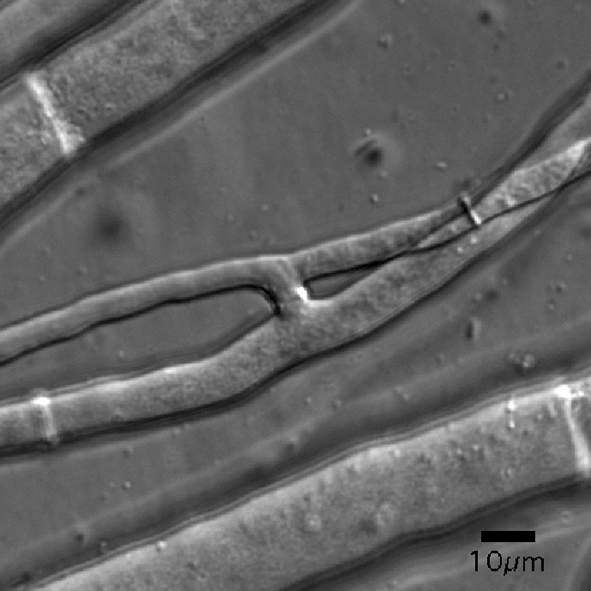
DIC image of two connected fusion hyphae through which cytoplasm is flowing. These fused hyphae were imaged while sandwiched between two layers of cellophane. Bar, 10 μm.

To verify that MSA-128 was defective in hyphal fusion, we developed a DIC-microscopy-based assay to identify intra-colonial hyphal fusion within growing colonies. Because intra-colonial cell fusion occurs rapidly, we found it difficult to routinely observe the cell fusion event, but it was quite easy to identify connected fusion hyphae that exhibited cytoplasmic continuity. This was done by observing the flow of cytoplasmic organelles as they moved from one vegetative hypha, through the connected fusion hyphae, into the second vegetative hypha. Fig. 1 shows two fusion hyphae that have participated in hyphal fusion as visualized in our experimental setup with mycelium sandwiched between two cellophane layers. A short video clip of cytoplasmic flow through connected fusion hyphae is available as a supplementary file with the online version of this paper (Supplementary movie 1). We could easily identify joined fusion hyphae with cytoplasmic flow in wild-type colonies, but rarely identified connected fusion hyphae with accompanying cytoplasmic flow in the MSA-128 mutant.

**Fig. 2.**
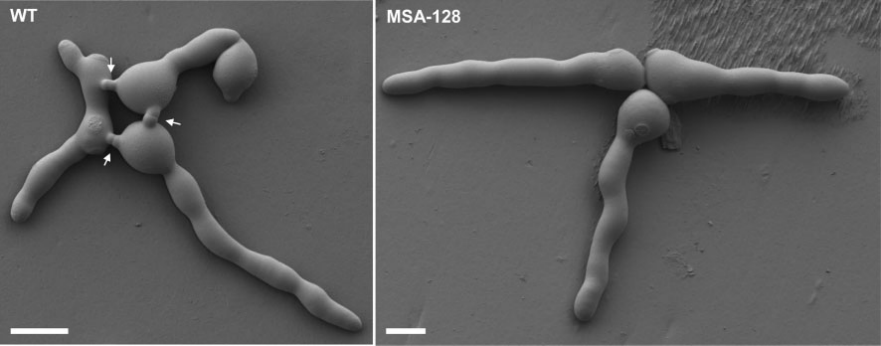
Low-temperature scanning electron microscopy of germinating conidia of the wild-type (WT) and *ham-5* mutant (MSA-128), showing that the mutant is unable to produce conidial anastomosis tubes (indicated by arrows for the wild-type). Bars, 10 μm.

We used a second microscopy-based assay to further characterize cell fusion in MSA-128. Conidia and germ tubes form specialized hyphae called conidial anastomosis tubes (CATs) that function in fusing conidal germlings during the early phase of colony establishment (Roca *et al.*, 2005a, b; Read *et al.*, 2009, 2010). Examination of germinating MSA-128 conidia by low-temperature scanning electron microscopy showed that the mutant conidia did not form CATs, while CAT formation was easily observed with wild-type conidia (Fig. 2). This lack of CAT formation in MSA-128 was verified by the light microscopic examination of hundreds of mutant germlings. We conclude that MSA-128 is unable to participate in CAT-based cell fusion events, as well as being defective in hyphal-based cell fusion in the growing colony.

**Fig. 3.**
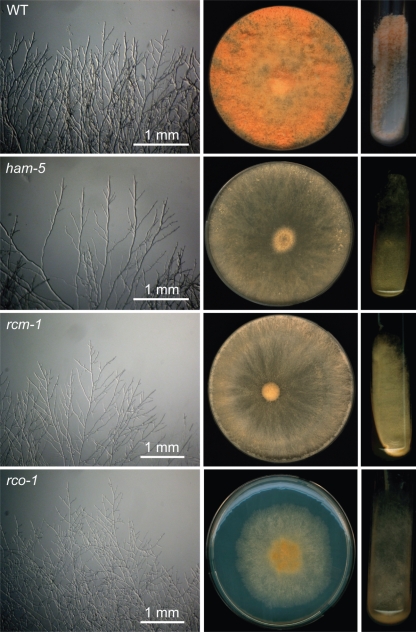
Morphology of the wild-type (WT) and hyphal fusion mutants showing growing hyphal tips at the colony periphery, growth on agar plates, and growth on agar slants. Photographs from the MSA-128 (*ham-5*), PB-S8 *(rcm-1*), and *rco-1^rip^* mutants are shown. Note that the mutant isolates produce conidia over the entire slant while the wild-type produces conidia in abundance at the top of the slant.

In addition to being unable to participate in hyphal fusion, MSA-128 has a number of other phenotypic characteristics. It grows more slowly than does its wild-type parent (at 25 °C the colony extension rate for MSA-128 was 1.2–1.4 mm h^−1^ as compared with 2.5–2.7 mm h^−1^ for its parental strain), and produces a less dense hyphal network than the wild-type. MSA-128 produces a different pattern of conidia formation on an agar slant (Fig. 3). Wild-type cells rapidly grow throughout the slant and produce an abundant array of aerial hyphae at the top of the slant. The aerial hyphae give rise to conidia, which in the slants inoculated with wild-type are located at the top of the slant. In MSA-128, short aerial hyphae are generated over the entire surface of the slant, and the conidia are produced as a ‘carpet’ over the whole area of underlying vegetative hyphae. Another phenotypic characteristic of MSA-128 is that the mutant is unable to produce protoperithecia, the immature female mating structures used in the sexual phase of the Neurospora life cycle. Thus, MSA-128 is unable to function as a female in the sexual phase of the life cycle and is therefore female infertile. However, crosses in which MAS-128 is used as the male (conidial) partner are fully fertile.

### The female-infertile PB-S8 mutant is hyphal fusion defective

Based on the observation that many hyphal fusion mutants are unable to form protoperithecia, we decided to examine protoperithecia-defective mutants for their ability to participate in hyphal fusion. The Fungal Genetics Stock Center has a number of such mutants in its collection, deposited by investigators over the past 5 decades (http://www.fgsc.net). We ordered some of these protoperithecia-defective mutants from the FGSC, and used the microscope-based cell fusion assay described in Methods to determine whether these mutants were defective in hyphal fusion. Among the hyphal fusion mutants identified was the PB-S8 mutant (FGSC 3086). This mutant was isolated by Johnson (1978) in a UV mutagenesis and screening procedure designed to identify mutants affected in female development. We were unable to find evidence of hyphal fusion events in our microscopic analysis of PB-S8, and this strain shared the other phenotypic characteristics we observed in MSA-128. In addition to being unable to participate in hyphal fusion within the growing colony and protoperithecia production, the PB-S8 mutant exhibited a reduced rate of hyphal extension (1.4–1.6 mm h^−1^ as compared to 2.5–2.5 mm h^−1^ for the wild-type) and produced conidia as a carpet over the surface of a slant, instead of the abundance of conidia at the top of the slant which is characteristic of the wild-type (Fig. 3). To further characterize the PB-S8 mutant, we used DIC microscopy to look for CATs and found that this mutant was unable to form them. Thus, based on the lack of CATs and fusion hyphae in the mature colony, we concluded that PB-S8 is hyphal fusion defective.

### Identification of the *ham-5, rcm-1* and *rco-1* genes as hyphal fusion genes

To identify the genes that were mutated in MSA-128 and PB-S8, we used a mapping and sequencing strategy. Because MSA-128 and PB-S8 are female infertile, all of the mapping experiments were done using them as the male partner in the mapping experiments. We had previously used a mapping and sequencing strategy to show that a mutation in the *mnt-1* gene, which encodes a putative *α*1,2-mannosyltransferase, was responsible for a colonial growth phenotype (Bowman *et al.*, 2005). We first mapped the mutations using classical Neurospora mapping techniques. We determined that the mutation in MSA-128 was located within 5 centimorgans of the *arg-12* gene on linkage group II of the *N. crassa* genetic map. Mapping experiments to determine the location of the mutation in PB-S8 showed that this gene is located between the *arg-5* and *arg-12* genes on linkage group II. The mutation mapped 13 centimorgans to the right of *arg-5* and 12.5 centimorgans to the left of *arg-12*.

To determine which gene had been mutated in MSA-128 to create the mutant phenotype, we examined the sequenced genome in the region defined by the mapping and identified 52 genes in that region that might have the relevant mutation. Based on the *N. crassa* genomic DNA sequence available at the Broad Institute (http://www.genome.wi.mit.edu; Dunlap *et al.*, 2007), we designed primers and PCR amplified the coding regions of these 52 genes from the MSA-128 genomic DNA. These amplified MSA-128 genomic DNAs were sequenced, and the sequences were compared with the published genomic DNA sequence. The *arg-12* isolate used to generate the MSA-128 mutant is closely related to the strain that was sequenced by the Broad Institute, and so a direct comparison with the published sequence was possible. We found two mutations within the 52 sequenced genes. One of these was in a ‘wobble-base’ position in the NCU01613 gene. Since this mutation would not affect the encoded protein, it is very unlikely to be responsible for the mutant phenotype. The second mutation was a deletion of a single base (T in the coding strand) in the NCU11925 gene, which encodes a 1686 amino acid protein. The deletion occurs within a phenylalanine codon (TTT) (codon 1379) and changes the reading frame. The deletion of a T from a sequence with two or more adjacent Ts is often associated with UV mutagenesis. UV irradiation is known to cause the formation of thymine dimers, and the replication of thymine-dimer-containing DNA can lead to a T/A base-pair deletion or a base substitution. The frameshift gave a stop codon 32 amino acids after the deletion to produce a truncated protein that lacked the C-terminal 308 amino acids. We concluded that this mutation is most probably responsible for the observed phenotype, and that NCU11925 is likely to be a gene involved in vegetative hyphal fusion. We named this gene *ham-5* (hyphal anastomosis mutant gene 5).

The NCU11925 gene has putative introns near the N-terminus of the encoded protein, and depending on how these introns are removed, a number of different translational products could be generated. To determine what the correct intron boundaries are, we used an RT-PCR approach to amplify the 5′ region of the mRNA, and analysed the PCR product by sequencing. The results demonstrated that the *ham-5* gene gives rise to a transcript encoding a protein with 1686 amino acids, as given for the NCU11925 gene on the Broad Institute website. The encoded protein contains two putative WD40 domains near the N-terminus, which suggests it might participate in protein–protein interactions. The putative WD40 domains are located between amino acids 72 and 111 and between amino acids 254 and 293. blast searches for *ham-5* homologues showed that the *ham-5* gene is highly conserved in many species of filamentous fungi. The HAM-5 homologue from *Podospora anserina* has been reported to function along with NADPH oxidase in directing the nuclear localization of a MAP kinase (Jamet-Vierny *et al.*, 2007).

To verify that the *ham-5* gene is required for hyphal fusion, we identified deletion mutants for this gene in the *N. crassa* genome project. The knockout deletion mutants (FGSC 15045 and FGSC 15046) have the entire NCU11925 coding region deleted. These mutants were obtained from the Fungal Genetics Stock Center and characterized. They were completely defective in intra-colonial hyphal fusion, and showed all of the other phenotypes that we identified in MSA-128 (reduced growth rate, altered conidiation pattern, protoperithecium-defective, and an inability to form CATs). Using PCR primers to amplify the disrupted gene, we verified that the *ham-5* gene was disrupted by the insertion of the hygromycin-resistance cassette used in creating the deletion mutants. We also demonstrated that the mutant phenotypes co-segregated with the disrupted gene, as defined by hygromycin resistance. Based on the data from MSA128 and the deletion mutant, we concluded that the *ham-5* gene is required for vegetative hyphal fusion and that all of the phenotypes we have described are due to mutations in this gene.

We used the same approach to identify the mutation with the PB-S8 mutant. We PCR amplified and sequenced 59 genes in the region defined as containing the PB-S8 mutation. Among the 59 genes sequenced, we identified a single mutation, found in the NCU06842 gene, which encodes a 918 amino acid protein. This mutation changed codon 339 from a leucine codon (TTA) to a stop codon (TGA). Similar to the situation with MSA128, the mutation in PB-S8 is consistent with the formation of a thymine dimer during UV mutagenesis. The NCU06842 gene encodes a tetratricopeptide-repeat-containing protein, and is a homologue of the yeast *SSN6* gene. A literature search indicates that a mutation in this gene was previously identified and reported on by Kim & Lee (2005), who designated it as the *rcm-1* (regulator of conidiation and morphology-1) gene. Kim & Lee (2005) generated the *rcm-1* mutant through the RIP mutational process, and characterized it as having a reduced growth rate, and being affected in both the sexual and asexual phases of the life cycle. Specifically the *rcm-1^rip^* mutant was female infertile because it was unable to generate protoperithecia, and produced conidia from short aerial hyphae. These are the same characteristics that we identified in the PB-S8 mutant (Fig. 3). Kim & Lee (2005) did not examine whether their *rcm-1* mutant was able to participate in vegetative hyphal fusion. Our data demonstrate that *rcm-1* is also required for hyphal fusion. The PB-S8 mutant was isolated long ago by Johnson (1978), and referred to as a female-sterile (fs) mutant, but the mutation was not mapped or given a name. Thus, we will refer to the gene (NCU06842) as the *rcm-1* gene, the name given by Kim & Lee (2005).

The *N. crassa rcm-1* gene product is a close homologue of the yeast *SSN6* gene, whose protein product functions in regulating transcriptional activity as a component of a dimer with the yeast TUP1 protein. If the *N. crassa* RCM-1 functions in a similar manner, we would expect *N. crassa* deletion mutants in the *TUP1* gene homologue, *rco-1* (NCU06205) (Madi *et al.*, 1994; Yamashiro *et al.*, 1996) to be defective in hyphal fusion. Yamashiro *et al.* (1996) used the RIP phenomenon to generate a *N. crassa rco-1* mutant, which they extensively characterized. Their *rco-1^rip^* mutant (FGSC# 9511) and knockout mutations in which the *rco-1* gene was deleted (FGSC 11371 and 11372) were obtained from the Fungal Genetics Stock Center and characterized. These *rco-1* mutants had the same characteristics that we observed in the PB-S8 mutant (Fig. 3). Specifically, the *rco-1* mutant phenotype included a reduced growth rate (0.4 mm h^−1^ for the *rco-1^rip^* mutant), an inability to participate in intra-colonial hyphal fusion, a reduction in the formation of CATs, an altered conidiation pattern, and an inability to form protoperithecia. We conclude that the *rco-1* mutants are compromised in vegetative hyphal fusion. The fact that *rco-1* mutants are compromised in hyphal fusion lends additional strong supporting evidence for the role of *rcm-1* as a hyphal fusion gene.

### RCM-1, RCO-1 and HAM-5 are not required to activate the MAK-1 and MAK-2 signal transduction pathways

**Fig. 4.**
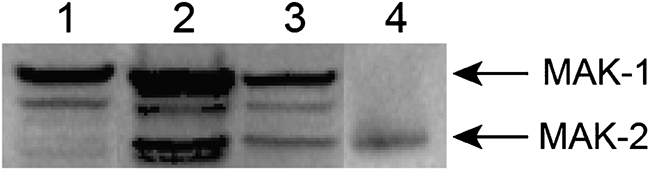
Western blot analysis of MAK-1 and MAK-2 phosphorylated activation sites in the *ham-5* knockout mutant. A single blot is shown in which a *mak-2* mutant (lane 1), the wild-type (lane 2), and *ham-5* knockout mutant (lane 3) were tested for the presence of activated MAK-1 and MAK-2. A positive control containing phosphorylated p42 MAP kinase (Erk2) is shown in lane 4. The arrows show the positions of MAK-2 (41 080 Da) and MAK-1 (51 580 Da).

*N. crassa* has three MAP kinase signal transduction pathways, characterized by the MIK-1/MEK-1/MAK-1, NRC-1/MEK-2/MAK-2 and OS-4/OS-5/OS-2 kinase cascades. The three pathways have been reported to be required for vegetative hyphal fusion (Maerz *et al.*, 2008; Pandey *et al.*, 2004; Park *et al.*, 2008; Roca *et al.*, 2005b). HAM-5, having a WD40 domain, is likely to be involved in protein–protein interactions. We considered the possibility that it might function as an upstream element in one of these MAP kinase pathways. We also considered whether the RCO-1 or RCM-1 transcription factors might be required for the expression of one or more proteins in these pathways. To examine the possibility that these hyphal fusion mutants might be defective in their ability to activate these pathways, we tested the ability of the mutants to phosphorylate the activation sites in MAK-1 and MAK-2. Phosphorylated MAK-1 and MAK-2 can be distinguished from each other on an SDS-PAGE gel based on their size differences: MAK-1 has a molecular mass of 51 580 Da while MAK-2 has a molecular mass of 41 080 Da. Primary antibody directed against the phosphorylated activation site of the human MAP kinase ERK1 (42 000/44 000 Da) has been previously shown to recognize both of these *N. crassa* MAP kinases (Maerz *et al.*, 2008; Pandey *et al.*, 2004). Cell extracts of wild-type and *ham-5* knockout mutant cells were prepared and subjected to Western blot analysis using primary antibody directed against the phosphorylated activation site, as described in Methods. Fig. 4 shows a representative experiment examining the phosphorylation status of MAK-1 and MAK-2 in the *ham-5* knockout mutant. The *ham-5* mutant extract contained phosphorylated MAK-1 and MAK-2, as did the wild-type control extract. Similar experiments demonstrated that the *rco-1* knockout mutant and *rcm-1* PB-S8 mutant were also able to phosphorylate MAK-1 and MAK-2. We conclude that the *ham-5*, *rco-1* and *rcm-1* mutants contain functional MAK-1 and MAK-2 signal transduction pathways.

## DISCUSSION

In this study we have identified three genes involved in regulating vegetative hyphal fusion in *N. crassa*. The first gene (*ham-5*) was initially identified in a screen for mutants defective in heterokaryon formation. We identified the second hyphal fusion gene (*rcm-1*) from a screen of female-sterile mutants in the FGSC mutant collection, performed because mutants lacking, or possessing defective, female sex organs (protoperithecia) are usually also defective in hyphal fusion (Read *et al.*, 2010). The *rcm*-1 gene is a homologue of the *S. cerevisiae SSN6* gene, whose product forms a dimeric trancription factor with TUP1. The third hyphal fusion gene (*rco-1*) that we have identified was the homologue of *TUP1*.

The *N. crassa ham-5* gene has close homologues in other filamentous fungi. The *P. anserina* homologue, *IDC1*, has been reported to function in directing the movement of a MAP kinase into the nucleus (Jamet-Vierny *et al.*, 2007). The *ham-5* gene encodes a 1686 amino acid protein with two putative WD40 domains; such domains function in mediating a variety of protein–protein interactions. WD40 domains have been found to mediate interactions between adaptor/regulatory modules involved in signal transduction, in interactions involved in pre-mRNA processing proteins, and in cytoskeleton assembly. The WD40 domain typically contains a GH dipeptide 11–24 residues from its N-terminus and the WD dipeptide at its C-terminus. The domain is 40 residues long; hence the name WD40. Between the GH and WD dipeptides lies a conserved core that serves as a stable propeller-like platform to which proteins can bind either stably or reversibly (Smith *et al.*, 1999). We have shown that the *ham-5* mutants are defective in CAT formation and cannot undergo hyphal fusion during colony initiation or in the mature colony. This suggests that the HAM-5 protein is needed for the production of CATs and possibly fusion hyphae, and might be part of a signalling pathway involved in generating cell-fusion-competent hyphae. We tested whether HAM-5 might be needed to activate the MAK-1 and/or MAK-2 MAP kinase signal transduction pathways, and found that the HAM-5 protein was not needed to phosphorylate the MAK-1 and MAK-2 activation sites. This suggests that HAM-5 does not function to activate these MAP kinase signal transduction pathways. Additional experiments will be needed to elucidate the function of the HAM-5 protein.

We also demonstrated that the *N. crassa rco-1* and *rcm-1* genes are required for CAT formation and vegetative hyphal fusion. The *S. cerevisiae* homologues of these genes encode the dimeric TUP1/SSN6 transcription factor (Kim & Lee, 2005; Yamashiro *et al.*, 1996). We therefore hypothesize that transcription directed by *rco-1 and rcm-1* is needed for hyphal fusion. The TUP1/SSN6 complex is a general repressor of transcription in yeast that functions in regulating growth and development (Keleher *et al.*, 1992; Redd *et al.*, 1997).

Hyphal fusion is an important process in the life cycles of filamentous fungi (Read *et al.*, 2010). All fusion mutants so far described have a pleiotropic phenotype. Common features of hyphal fusion mutants in *N. crassa* have been that they are also compromised in sexual development, have reduced hyphal growth rates and exhibit conidiation defects. Altered production of the female mating structure in hyphal fusion mutants has also been documented in the closely related fungus *Sordaria macrospora* (Engh *et al.*, 2007; Poggeler *et al.*, 2006), and observed in *Aspergillus nidulans* (Wei *et al.*, 2003). An interesting question is whether defective vegetative hyphal fusion is the basis of all of these other defects, or whether there are other roles for these hyphal fusion genes. It is known that some of the proteins involved in hyphal fusion (e.g. MAK-2 and SO) have other roles in *N. crassa*. The MAP kinase MAK-2, for example, is likely to be involved in the pheromone response pathway in *N. crassa*, similar to its orthologue Fus3 in *S. cerevisiae* (Dohlman & Thorner, 2001). Another protein (SO) involved in hyphal fusion in *N. crassa* (Fleissner *et al.*, 2005, 2009) also seems to have a role in septal pore plugging (Fleissner & Glass, 2007).

It is also possible that the defective protoperithecium production and altered conidiation pattern phenotypes of hyphal fusion mutants may be influenced by the lack of intra-colonial fusion reducing the movement of cytoplasmic resources to sites of protoperithecium formation and conidiation. When protoperithecium production is stimulated by nitrogen deprivation (Davis & DeSerres, 1970), there is a requirement for cytoplasmic resources to be redistributed through the interconnected vegetative hyphal network to the developing protoperithecia. In the absence of hyphal fusion, the interconnectedness of the hyphal elements is compromised, and as a result the fungus may be unable to efficiently move cytoplasmic resources into the developing protoperithecia. Similarly, the altered conidiation pattern we observe in all of our fusion mutants may be explained as arising from the hyphal fusion defect. Conidiation occurs where the CO_2_ levels are lowest and the oxygen levels are highest (Sargent & Kaltenborn, 1972), which in a colony growing on an agar slant in a test tube is at the top of a slant. In a conidiating wild-type colony, cytoplasmic resources are redistributed from other locations throughout the colony to the hyphae at the top of the slant. This supports the growth of aerial hyphae and the formation of conidia at that location. We hypothesize that in the absence of hyphal fusions, the redistribution of cytoplasmic resources is compromised, and instead of producing conidia on long aerial hyphae at the top of the slant, short aerial hyphae are produced throughout the entire colony, and conidia are produced over the entire surface of the slant.

## Abbreviations

- CAT, conidial anastomosis tube
- DIC, differential interference contrast
- RIP, repeat-induced point mutation
